# Supplementation of Korean Red Ginseng improves behavior deviations in animal models of autism

**DOI:** 10.3402/fnr.v60.29245

**Published:** 2016-02-01

**Authors:** Edson Luck T. Gonzales, Jong-Hwa Jang, Darine Froy N. Mabunga, Ji-Woon Kim, Mee Jung Ko, Kyu Suk Cho, Geon Ho Bahn, Minha Hong, Jong Hoon Ryu, Hee Jin Kim, Jae Hoon Cheong, Chan Young Shin

**Affiliations:** 1 Department of Neuroscience, School of Medicine, Konkuk University, Seoul, Korea; 2 Neuroscience Research Center, IABS, Konkuk University, Seoul, Korea; 3 KU Open Innovation Center, Konkuk University, Seoul, Korea; 4 Department of Dental Hygiene, Hanseo University, Seosan, Korea; 5 Department of Neuropsychiatry, School of Medicine, Kyung Hee University, Seoul, Korea; 6 Department of Psychiatry, School of Medicine, Dankook University Hospital, Cheonan, Korea; 7 Department of Oriental Medicine, Kyung Hee University, Seoul, Korea; 8 Department of Pharmacy, Sahmyook University, Seoul, Korea

**Keywords:** *Panax ginseng*, nutraceutical, autistic behaviors, Korean Red Ginseng, prenatal VPA exposure

## Abstract

**Background:**

Autism spectrum disorder (ASD) is heterogeneous neurodevelopmental disorders that primarily display social and communication impairments and restricted/repetitive behaviors. ASD prevalence has increased in recent years, yet very limited therapeutic targets and treatments are available to counteract the incapacitating disorder. Korean Red Ginseng (KRG) is a popular herbal plant in South Korea known for its wide range of therapeutic effects and nutritional benefits and has recently been gaining great scientific attention, particularly for its positive effects in the central nervous system.

**Objectives:**

Thus, in this study, we investigated the therapeutic potential of KRG in alleviating the neurobehavioral deficits found in the valproic acid (VPA)-exposed mice models of ASD.

**Design:**

Starting at 21 days old (P21), VPA-exposed mice were given daily oral administrations of KRG solution (100 or 200 mg/kg) until the termination of all experiments. From P28, mice behaviors were assessed in terms of social interaction capacity (P28–29), locomotor activity (P30), repetitive behaviors (P32), short-term spatial working memory (P34), motor coordination (P36), and seizure susceptibility (P38).

**Results:**

VPA-exposed mice showed sociability and social novelty preference deficits, hyperactivity, increased repetitive behavior, impaired spatial working memory, slightly affected motor coordination, and high seizure susceptibility. Remarkably, long-term KRG treatment in both dosages normalized all the ASD-related behaviors in VPA-exposed mice, except motor coordination ability.

**Conclusion:**

As a food and herbal supplement with various known benefits, KRG demonstrated its therapeutic potential in rescuing abnormal behaviors related to autism caused by prenatal environmental exposure to VPA.

Autism spectrum disorders (ASD) is a range of neurodevelopmental disorders that generally characterize social communication and social interaction difficulties accompanied by restrictive and repetitive behaviors (1). ASD continues to gain much attention in the society because of its increasing diagnoses and heterogeneity but still has unexplained proposed etiologies and limited therapeutic entities (2). Thus, many researchers, including us, have given much focus on how to uncover this formidable condition by finding the possible etiologies, tracing the pathologic mechanisms and discovering therapeutic treatments of ASD in many animal models (3–13). Most importantly, the current medication used for the treatment of ASD is only symptomatic and limited to the alleviation of repetitive behaviors, as well as other comorbid symptoms such as depression, seizure, aggression, and sleep or gastrointestinal disturbances. While a tough race continues for the development of small molecular therapeutic entities against ASD, one possible approach is to investigate the beneficial effects of functional food items as well as herbal medicines.

Ginseng, belonging to the genus *Panax*, is a popular herbal plant widely used for its variety of effects in both traditional and research-based medicines. For many years, ginseng gained a lot of attention everywhere for its adaptogenic, aphrodisiac (14) [including treatment of erectile dysfunction (15)], anticarcinogenic (16–18), antioxidant (17, 19, 20), and antiobesity (21) properties, among others, in its several ginsenoside compounds that can be found extensively in Korean Red Ginseng (KRG). Red ginseng is a sun-dried and steamed form of the harvested root of the plant. Choi published an ample review about the characteristics and pharmacological and medicinal components of ginseng (22).

In addition to its known benefits, studies of KRG related to the central nervous system (CNS) have been gaining knowledge as well [see for review the works of Kim, HJ (23)]. In both human and animal studies, KRG, in different preparations and extractions, showed considerable benefits in Alzheimer's disease (24), cerebral blood flow, inhibition of superoxide production (25), ischemic injury (26, 27) and learning and memory (28, 29), among others. Thus, it must be interesting to investigate the effect of KRG on neurodevelopmental disorders such as ASD and others.

Valproic acid (VPA) and other valproate products are known antiepileptic drugs (AED) (30) and are also used for maintenance treatment of manic type bipolar disorder (31) and migraine headache (32). Over the years, VPA used during pregnancy was found to cause varying degrees of cognitive deficits (33), birth defects (34, 35), and increased risk of autism in human offspring (36). Thus, VPA-exposed animal models have been widely used by many researchers including us to possibly explain the underlying pathologic pathways of ASD and apply existing and novel therapeutic entities for ASD based on the impaired pathway [see for review (37)].

VPA-exposed models of ASD show neural tube defects (observed as crooked tails), neurochemical alterations, and behavioral deficits such as social impairments (37). Previously, we also showed the preventive effect of chronic KRG treatment given at embryonic days 10–15 (E10–E15) from the deleterious effect of prenatal VPA exposure at E12 and rescued the neural tube defects, social impairments, and increased seizure susceptibility in offspring rats (13). These results paved a way to further study the therapeutic potentials of postnatal KRG treatment in VPA-exposed ASD animal models which could have a stronger clinical importance in a real-world situation. In this study, we asked whether long-term KRG treatment to offspring mice could alleviate the social and other behavioral impairments after *in utero* VPA exposure. The impact of the result of this study can lead to more promising therapeutic opportunities of KRG for the rising diagnosis but limited treatment of ASD clinically.

## Materials and methods

### Animals

**Fig. 1:**
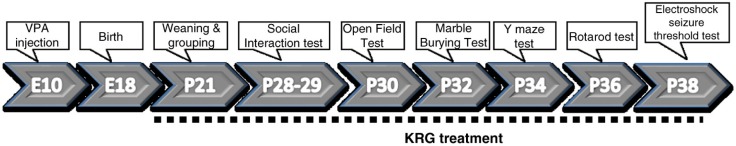
The experiment scheme shows the main step periods from *in utero* VPA exposure to long-term postnatal KRG treatment along with the behavior tests employed.

Animal treatment and maintenance were carried out in agreement with the Animal Care and Use Guidelines of Sahmyook University and Konkuk University, Korea, and in accordance with the 14th article of the Korean Animal Protection Law as well as the Principle of Laboratory Animal Care (NIH publication No. 85–23, revised 1985) (38). All efforts were made to minimize the number of animals as well as their suffering. Ten female ICR mice at gestational day 5 were purchased from OrientBio (Gyeonggi-do, Korea). They were maintained on a 12:12-h circadian cycle with lights on at 07:00 and off at 19:00, at a constant temperature (22±2°C) and humidity (55±5%). Treatments were administered according to the schedule (Fig. 1), and behavior tests were carried out in designated rooms. Subjects were given ample time to rest in between experiments.

### Subcutaneous injection of VPA to pregnant mice

Sodium VPA (Sigma, St. Louis, MO) was dissolved in 0.9% saline at a concentration of 50 mg/mL, pH 7.3. Pregnant mice were subcutaneously injected with either 300 mg/kg of VPA or saline solution in the loose skin area of the neck on gestational day 10 (E10). VPA-exposed male offspring were randomly assigned in groups to distribute each litter equally to different treatment conditions.

### KRG preparation and treatment of male offspring mice

The KRG extract used in this study was manufactured from the roots of a 6-year-old fresh ginseng, *Panax ginseng* Meyer, harvested in the Republic of Korea by Korea Ginseng Corporation, Seoul. Red ginseng was made and extracted as described previously (13) in a standardized method from previous studies (39–41). From the VPA- and saline-treated mothers, male offspring were randomly selected from each litter to be assigned in a designated group. The groups include the control, VPA, VPA+KRG in 100 mg/kg, and VPA+KRG in 200 mg/kg. Each group was given daily oral administrations of either distilled water or KRG solution from P21 and continued until the termination of all experiments. KRG extracts were freshly prepared by dissolving in distilled water before administration. All in all, a total of 10 mice in each group (Control, VPA, VPA+KRG100, VPA+KRG200) were utilized for behavior testing. Behavior assessment of each group started at P28 until P40 with the following tests described below and as summarized in Fig. 1.

### Three-chamber sociability and social novelty preference tests

The modified sociability and social novelty preference tests as described previously in our lab (12) was originally established by Moy et al. (42). The test was performed in a three-chamber rectangular cage (23×40×22 cm in each compartment). Two 10 cm^2^ openings provided access to the three chambers. The central compartment was the starting area of each trial and both the side compartments contain a round wire cage of 10 cm diameter wherein conspecific stranger mice were put (during social novelty preference test) and one left empty during sociability test. Stranger mice were of the same age and gender and had never encountered the subject mice previously. Stranger mice were habituated to the wire cage twice for 2 days before the actual test. Testing period takes approximately 30 min including 5-min habituation, 10-min sociability test, 10-min social novelty preference test, and few minutes for cleaning, consecutively.

Sociability tests determine the social interaction of subject mice to the stranger mouse in the wire cage (SM) versus the empty cage (EC). The duration in each compartment was tracked by EthoVision software (EthoVision 3.1, Noldus Information Technology, the Netherlands), and sniffing duration to each wire cage was measured by ‘blind observers’ 2 m away from the box. Sociability index (SI; SM compartment duration/EC compartment duration) was further calculated. Different mice groups were tested in every consecutive trial to avoid time difference bias.

Immediately after a sociability test, social novelty preference test began to assess the preference subject mouse to explore a novel mouse/object over an already familiar one. The previous SM had become the familiar mouse (FM), while a novel stranger mouse (NM) was introduced in the previously empty wire cage. The subject mouse was put back in the central compartment to begin the test. The duration in each compartment, social preference index (NM compartment duration/FM compartment duration), and sniffing duration in each cage were measured in the same way as the sociability test. At the end of each complete trial, animals were returned to their respective cages and the box surface was wiped and cleaned with 70% ethanol solution. Tests were performed between 9:00 and 17:00 hours. EthoVision software connected through an overhead camera tracked the movement of each subject in every compartment.

### Open-field test

An open-field test was performed to assess the spontaneous locomotor activity of mice. Mice were exposed for the first time in an open box (42×42×42 cm). Five boxes are available to test five animals simultaneously for video recording and behavior tracking using EthoVision software. The animals were allowed to habituate for 5 min after their introduction to the central area, and locomotor behavior was measured for 20 min. The distance moved, and movement duration was calculated. The surfaces of each box were cleaned and wiped with 70% ethanol at the end of each trial.

### Marble-burying test

This test was performed to assess the stereotyped and repetitive behaviors of VPA-exposed mice and was described previously, which we slightly modified in our study (43). Twenty marbles (15 mm diameter) were evenly arranged (5×4) on the bedding surface after 10-min habituation to the plastic home cages (12×26×12 cm) filled with 5-cm deep and lightly pressed corncob bedding. Each mouse was put back in the marble-filled cage and left for 20 min. Afterward, mice were carefully and swiftly removed in each cage, and the number of marbles covered with bedding up to more than 50% its surface was counted by a ‘blind observer’.

### Y-maze test

Spatial working memory through spontaneous alternations can be assessed in a Y-maze test (44). This procedure is based on the knowledge that a normal animal (the subject) has an innate inclination to visit an arm, which was not previously explored (45). The maze was made of black polyvinyl chloride with three arms (5 cm×35 cm×10 cm) forming a Y shape in equal angles. Each mouse was introduced in one of the arms facing the central area. Total entries and spontaneous alternations (total alternations/[total arm entries – 2]×100) of arm entries were recorded for 8 min (46). The animals were removed, and the surfaces were cleaned with 70% ethanol at the end of each trial.

### Rotarod test

The rotarod apparatus (Ugo Basile Srl, Comerio, Italy) is made of a corrugated drum (3 cm diameter) divided into five compartments by round plastic walls. In this study, we employed the fixed speed rotarod as previously described (47), which we slightly modified. The rotation speed was set at 36 rpm constantly, and animals were trained in this situation for 5 min a day prior to the actual test. The latency to fall and falling frequency were recorded during the 20-min trial duration in each mouse.

### Measurement of electroshock seizure threshold

As previously described (12, 13, 48), electroshock seizure threshold was measured in 5-week old mice with slight modifications. The seizure was induced by a current stimulator (ECT unit, Ugo Basile, Italy) attached as ear clips or auricular electrodes, and the full-scale seizure was observed by full hind limb extension. Convulsive current 50 (CC_50_), as defined by convulsion in 50% of animals, was determined through ‘staircase’ procedure and calculated by the Litchfield–Wilcoxon II method (49). The current settings were at 100 Hz frequency, 0.5 ms pulse width, 2 sec shock duration with a primary current set at 20 mA. One seizure induction was given to each mouse and if it showed full hind limb extension, the next mouse was induced with a current 2 mA lower than the previous setting. Otherwise, the current was increased by 2 mA if no full seizure response was observed.

### Statistical analysis

All data were presented as the means±standard error of mean (SEM), and statistics were analyzed using a non-parametric Kruskal–Wallis test followed by Dunn's multiple comparison test for all pairwise comparisons. Unpaired Student's *t-*test was also used to compare the difference of responses in behaviors between the control and the VPA-exposed mice. Statistical significance was set to a *p* value of less than 5% (*p*<0.05). All analyses were calculated using GraphPad Prism version 5 software.

## Results

### Long-term KRG treatment improved the VPA-induced impairments in sociability of mice offspring

**Fig. 2:**
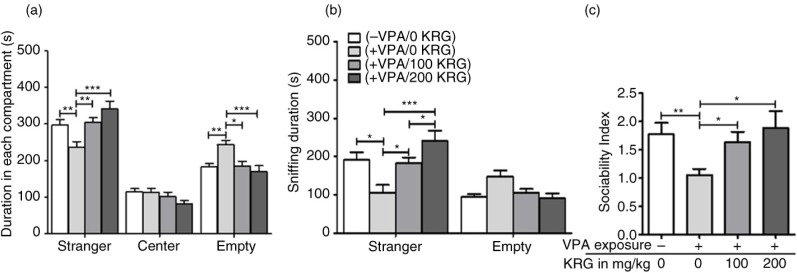
Effects of long-term KRG treatment on impaired sociability of VPA-exposed mice in the three-chamber apparatus by measuring the duration in each compartment (a), sniffing duration in the wire cages with or without stranger mouse (b), and the social preference index by comparing the duration between the stranger and empty compartments (c). Bars indicate the mean±SEM. *n*=10 mice per group. *p<0.05, **p<0.01, and ***p<0.001.

VPA injection at E10 in pregnant ICR mice has presented a socially impaired phenotype in the male offspring. Figure 2a revealed that the VPA-exposed mice had lower stay duration in the SM compartment compared to control (*p*<0.01). Interestingly, KRG treatments normalized the VPA-induced impairment to control levels (*p*>0.05) and were significantly different to VPA-only group (100 mg/kg, *p*<0.01; 200 mg/kg, *p*<0.001). Conversely, duration in the empty wire cage compartment was higher among the VPA-exposed mice as compared to the control (*p*<0.01) and the KRG-treated VPA-exposed mice groups (100 mg/kg, *p*<0.05; 200 mg/kg, *p*<0.001). Meanwhile, time spent in the central area did not differ among groups.

In Fig. 2b, we further compared the sniffing behavior (quantified by time) of each group to the SM or the empty wire cage (50). Results showed a lower sniffing duration of the VPA-exposed mice than that of the SM as compared to the control (*p*<0.05), while KRG treatment rescued this impairment (100 mg/kg, *p*<0.05; 200 mg/kg, *p*<0.001) to control level (*p*>0.05). Sniffing duration of the VPA-exposed mice in the empty wire cage showed a trend that was slightly higher than the control and the KRG treated groups (*p*>0.05). The 200 mg/kg dose of KRG also had a significantly higher sniffing duration in the SM than 100 mg/kg dose (*p*<0.05) but not against the control group (*p*>0.05). SI further confirmed that the VPA-exposed mice have a significantly lower index than the control (*p*<0.01) and the KRG-treated groups (*p*<0.05) (Fig. 2c). Taken together, the results suggest that prenatal VPA exposure can cause a significant decrease in sociability behavior of mice offspring and long-term treatment of KRG using 100 mg/kg and 200 mg/kg dosages, which could rescue this impairment to control levels.

### Long-term KRG treatment improved the VPA-induced impairments in social novelty preference in mice offspring

**Fig. 3:**
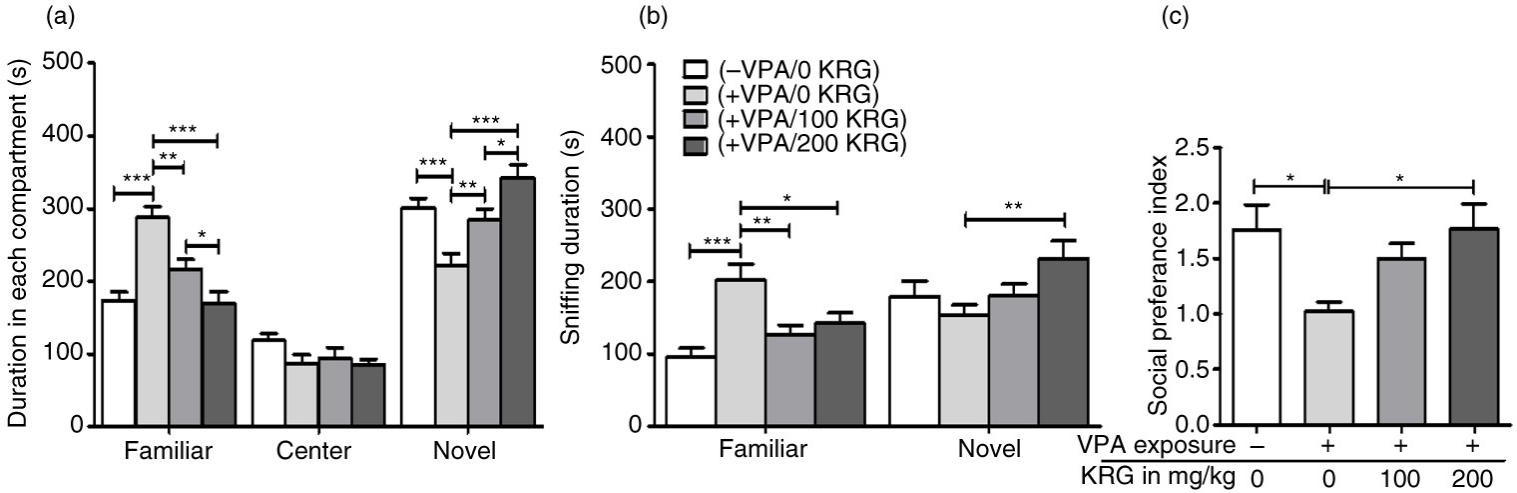
Effects of long-term KRG treatment on impaired social preference of VPA-exposed mice in the three-chamber apparatus by measuring the duration in each compartment (a), sniffing duration in the wire cages with novel or familiar mouse (b), and the sociability index by comparing the duration between the novel and familiar compartments (c). Bars indicate the mean±SEM. *n*=10 mice per group. *p<0.05, **p<0.01, and ***p<0.001.

Social novelty preference test immediately followed the sociability test for each mouse. As shown in Fig. 3a, duration in the FM (previously SM) compartment was higher for the VPA-exposed mice as compared to the control (*p*<0.001) and the KRG-treated VPA-exposed mice (100 mg/kg, *p*<0.01; 200 mg/kg, *p*<0.001). In addition, duration in the novel mouse compartment (previously EC) was significantly lower for the VPA-exposed mice than the control (*p*<0.001) and the KRG-treated VPA-exposed mice (100 mg/kg, *p*<0.01; 200 mg/kg, *p*<0.001). Time spent in the central compartment did not differ among all groups. The result also showed that VPA-induced social preference impairment was normalized to control levels by long-term KRG treatment. Interestingly, there was a significant difference between the KRG-treated VPA-exposed mice groups, with the 200 mg/kg dose group having a higher duration in novel mouse compartment and lower duration in FM compartment than the 100 mg/kg dose group (*p*<0.05).

In accordance with the duration in the compartments, sniffing duration may further show a more sensitive and ethological measure of social preference in mice (50) (Fig. 3b). The VPA-exposed mice showed a higher sniffing duration to the FM than the control (*p*<0.001) and the KRG-treated VPA-exposed mice groups (100 mg/kg *p*<0.01; 200 mg/kg, *p*<0.05). On the other hand, sniffing duration of the VPA-exposed mice to the novel mouse significantly differed only against the 200 mg/kg KRG-treated VPA-exposed mice group (*p*<0.01) but not against the control (*p*>0.05) or the 100 mg/kg KRG-treated VPA-exposed mice group (*p*>0.05). Social novelty preference index (SPI) was evidently lower in the VPA-exposed mice than the control group (*p*<0.05) and the 200 mg/kg KRG-treated VPA-exposed mice group (*p*<0.01) but not in the 100 mg/kg KRG-treated VPA-exposed mice group (*p*>0.05) (Fig. 3c), which was encouraging. Overall, we have quantified that the VPA-exposed mice showed an impaired social novelty preference and long-term KRG treatment can further rescue this impairment.

### Long-term KRG treatment alleviated the hyperactivity phenotype in the VPA-exposed mice offspring

**Fig. 4:**
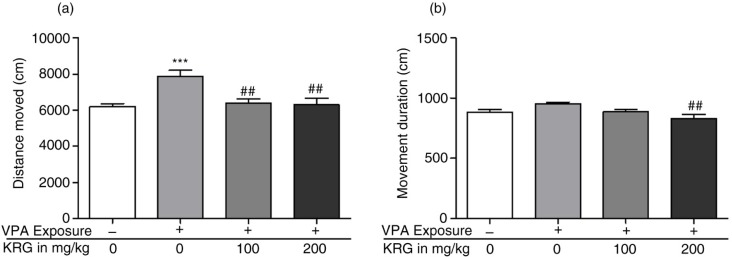
Effects of long-term KRG treatment on hyperactivity phenotype of VPA-exposed mice in the open-field apparatus by measuring the distance moved (a) and movement duration (b). Bars indicate the mean±SEM. *n*=10 mice per group. ***p<0.001 vs. control group and ^##^*p*<0.01 vs. VPA only group.

The VPA-exposed mice exhibit a hyperactive phenotype when compared to the control group as measured by the distance moved (Fig. 4a) in the open field (*p*<0.001). However, there was no significant difference between the VPA-exposed mice and the control mice in the movement duration parameter (Fig. 4b) (*p*>0.05). Interestingly, KRG treatment normalized the VPA-induced hyperactivity phenotype to control levels (100 mg/kg, *p*<0.01; 200 mg/kg, *p*<0.01) in the distance moved parameter (Fig. 4a). Furthermore, only the 200 mg/kg VPA dosage showed a significant decrease in the increased movement duration (Fig. 4b) of the VPA-exposed mice (*p*<0.01).

### Long-term KRG treatment alleviated the increased marble burying and normalized the decreased spontaneous alternation behavior in the VPA-exposed mice offspring

**Fig. 5:**
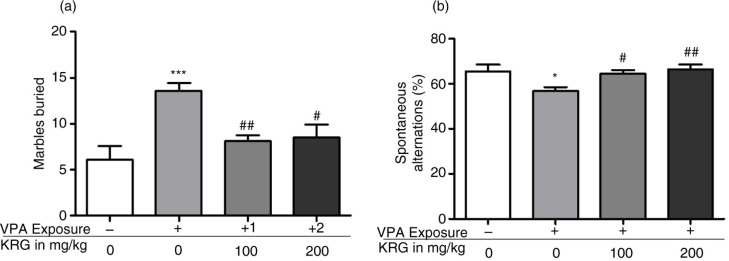
Effects of long-term KRG treatment on increased repetitive behavior of VPA-exposed mice in the marble-burying test (a) and impaired spontaneous alternations in the Y-maze test (b). Bars indicate the mean±SEM. *n*=10 mice per group. *p<0.05, ***p<0.01 vs. control group and ^#^*p*<0.05, ^##^*p*<0.01 vs. VPA only group.

In Fig. 5a, the VPA-exposed mice showed increased marble-burying behavior as compared to the control group (*p*<0.01). Interestingly, long-term KRG treatment decreased the burying behavior of the VPA-exposed mice (100 mg/kg, *p*<0.01; 200 mg/kg, *p*<0.05) to control levels. In addition, spontaneous alternation of the VPA-exposed mice was significantly lower than the control group (*p*<0.05) in the Y-maze test (Fig. 5b). Remarkably, KRG treatment of the VPA-exposed mice normalized the decreased spontaneous alternations (100 mg/kg, *p*<0.05; 200 mg/kg, *p*<0.01) to control levels.

### Tendency of motor balance and coordination impairment in the VPA-exposed offspring

**Fig. 6:**
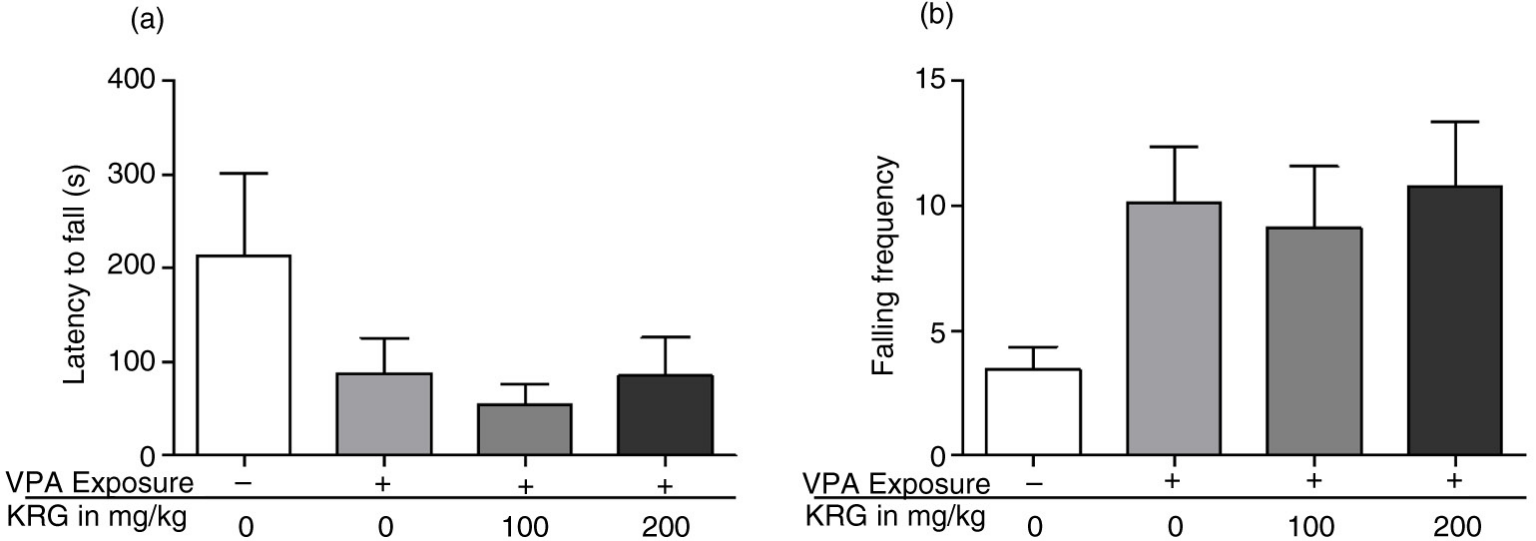
Effects of long-term KRG treatment on impaired motor coordination and balance of VPA-exposed mice in the fixed speed rotarod apparatus by measuring the latency to fall (a) and falling frequency (b) from the rotarod. No significance was observed. Bars indicate the mean±SEM. *n*=10 mice per group.

As shown in Fig. 6, there was a tendency but no significant difference between the VPA-exposed mice and the control group in the latency to fall and falling frequency performance in the rotarod test (*p*>0.05). Furthermore, there was no effect found in the KRG treatment in the overall rotarod performance.

### Long-term KRG treatment rescued the decreased seizure threshold in the VPA-exposed mice offspring

**Fig. 7:**
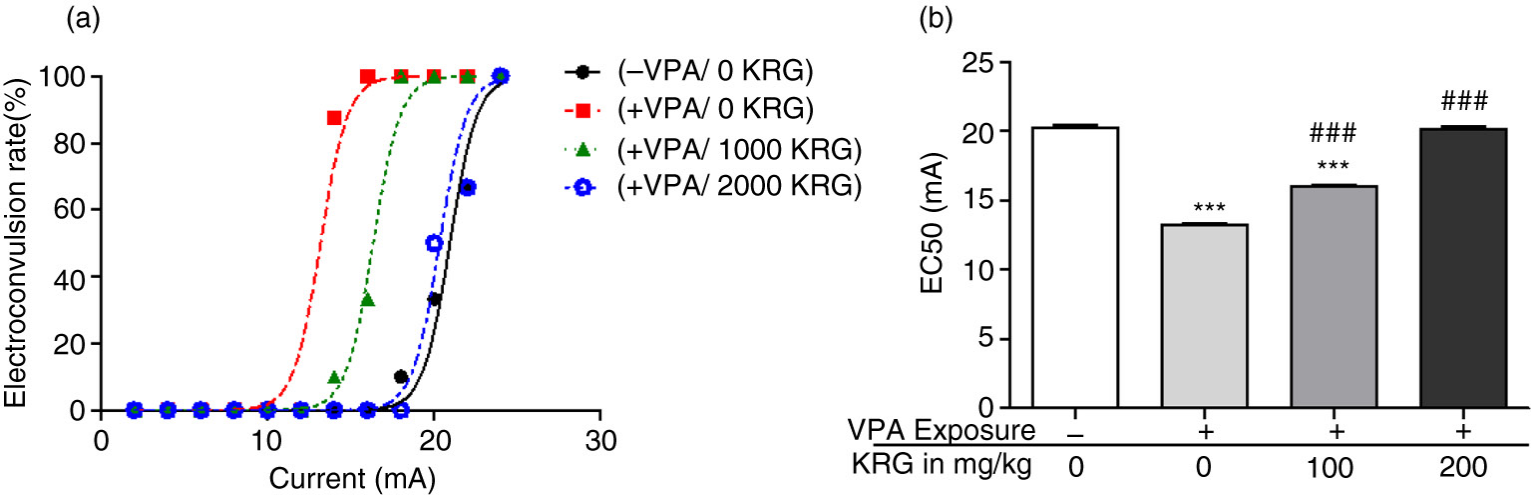
Effects of long-term KRG treatment on decreased electroshock seizure threshold of VPA-exposed mice by determining the convulsive current (CC_50_) using the ‘staircase’ method and calculating the electroconvulsive rate in percentage (a) and mean EC50 current threshold (b). Data are expressed in the non-linear fit graph (a) and a bar graph that indicates the mean current of seizure response of each group (b). *n*=10 mice per group. ***p<0.001 vs. control group and ^###^*p*<0.001 vs. VPA-only group.

VPA-exposed mice showed a significantly lower threshold against electrically induced seizure as compared to control group (Fig. 7). Interestingly, however, KRG treatment dose dependently normalized this impairment to control levels with 200 mg/kg dosage being highly effective. Each point in Fig. 7a shows the percentage of rats that exhibited full hind limb extension response to the electric currents employed.

## Discussion

The present study reveals the potential therapeutic effects of KRG when treated postnatally in VPA-exposed mice offspring models of ASD. Here, we showed that long-term KRG treatment in VPA-exposed mice rescued the various behavioral impairments related to ASD including sociability and social preference, locomotor activity, marble burying, spontaneous alternation, and electroshock seizure threshold. The order of experiments was planned considering the validation of ASD symptoms and the degree of stress they can induce in animals. We first conducted the social test, as this is crucial to determine whether the VPA-induced mice show a validated core symptom of autism. We followed it up with open-field test for the measurement of locomotor activity and marble-burying test for repetitive behaviors based on their relevance to ASD and the complexity of the experimental methods. Y-maze and rotarod tests determine the variable symptoms accompanying the ASD in terms of spatial working memory and motor coordination, respectively. The experiments ended with the electroshock seizure threshold test, which was obviously most stressful to the animals.

KRG treatment was initiated on 3-week-old VPA-exposed mice and was given a duration of 7 days for treatment prior to the start of behavioral assessments. Furthermore, KRG treatment continued until all behavior tests were done. We considered this as long-term treatment of KRG, which showed therapeutic effects in previous studies with different pathologic conditions in mice (51, 52) and humans (53, 54). The KRG dosages that we employed in this study were based on their efficacy in alleviating the impairments found in our previous study (13). Indeed, these dosages are well within a range to show a therapeutic effect in the behavior of VPA-treated animals because other dosages, such as 50 mg/kg, did not show a robust efficacy in our preliminary study (data not shown).

To roughly depict the clinical scenario of the treatment regimen of KRG in autistic patients, we used juvenile mice to represent school-age and puberty stages in humans. Previous research studied the ages of mice in comparison to human life stages showing that a 1-month-old mouse is equivalent to a 12.5-year-old child (55). Thus, the age of mice when KRG treatment started (P21) was approximately equivalent to the beginning of the school-age stage in humans. Furthermore, the experimental period between P28 and P38 could be highly similar to the puberty stage of human development in which the treatment regimen was still ongoing. Thus, the treatment period in this study represents a clinical set-up where long-term treatment of KRG started at an age when a child is usually diagnosed with ASD.

The sociability and social novelty preference deficits that we found in this study are in agreement with previous results using rats (10, 12, 13) and mice models (56–59). One of the primary diagnostic criteria for ASD is social impairment (1), and we have presented the validity of using the VPA-exposed animal models of ASD in this study. Thus, we investigated whether KRG could rescue the ASD-related impairments brought about by prenatal VPA exposure. Indeed, the long-term administration of KRG revealed its promising therapeutic properties in ameliorating the social deficit found in VPA-exposed mice. This enlightens our previous question on whether KRG effect, on the prevention of the neural tube defect when given during pregnancy, is also directly related to the behaviors in animal offspring (13). Our present results remarkably showed that the effect of KRG in reversing the social impairments goes beyond structural protection as it rescued the socially impaired phenotype in the developmentally affected VPA-exposed mice. Ultimately, studying the brain of VPA-exposed mice after KRG treatments and behavioral improvements would be an important step in elucidating the effects of KRG in the brain structural and neurochemical levels.

Hyperactivity phenotype in VPA-exposed animal models is well known in studies using rats (6, 13). However, a previous study using mice did not find differences in locomotor activity between control and VPA-exposed groups (58), but it should be noted that the length of the trial was only 5 min in that study. In another study, no significant difference in locomotor activity for 30-min testing period was also observed when they tested the locomotor behavior of VPA-exposed and control animals from ages P56 to P64, while VPA exposure was given during E13 to pregnant mothers (60). In previous studies as well as our present study, we performed an open-field test for 20 min in juvenile mice where we found hyperactivity in the VPA-exposed mice group (59). ASD and its comorbid attention deficit hyperactivity disorder (ADHD)-like behavior, especially the hyperactivity and impulsivity phenotypes, are more noticeable in younger children. Thus, assessing the locomotor behavior of animal models at an earlier age would be appropriate. We suggest and validate that the VPA-exposed mice models of ASD also show hyperactivity compared with the rat models in previous studies (6, 13). Remarkably, long-term KRG treatment normalized the hyperactivity of the VPA-exposed mice to control levels, which are reminiscent of our previous reports demonstrating the anti-hyperactivity effects of KRG in an animal model of ADHD (61).

Other relevant symptoms observed in ASD are restrictive, repetitive behaviors. The VPA-exposed mice models were validated previously to show increased repetitive behavior as measured by self-grooming and marble-burying assays (60). On a positive note, we found an increased marble-burying behavior of the VPA-exposed mice in the present study. Of interest, long-term KRG treatment alleviated the increased marble-burying behaviors in the VPA-exposed mice. A role of mGluR antagonism on the alleviation of repetitive behaviors in the VPA-exposed mice models was previously demonstrated (60). In addition, we observed a dysregulation of mGluR pathways in the VPA-exposed rat models of ASD (62). It is therefore of great interest to investigate the effect of KRG related to glutamatergic functions in the brain, such as mGluR5 antagonism in the future investigation.

Working memory is known to be declined in children and adolescents with idiopathic autism and 22q11.2 hemizygosity as found in previous studies (63–65). Indeed, a genetic model of autism, known as the *Tbx1* heterozygous (HT) mice in which the mutated *Tbx1* gene is part of the chromosome region 22q11.2 in humans, has shown a decreased T-maze spontaneous alternation along with ASD-related social interaction and ultrasonic vocalization impairments (66). Interestingly, our VPA mice model of ASD also showed a decreased spontaneous alternation in the Y-maze paradigm. Thus, our VPA mice model agrees with the previous genetic model of ASD having impairments in working memory and social interaction. Furthermore, while the VPA-exposed mice show impaired Y-maze spontaneous alternations, KRG treatment reversed the condition to control levels. It must be interesting and revealing to study the working memory-related hippocampal region of the VPA-exposed mice and the effect of KRG in the pathologic process involved in ASD.

Previously, we have consistently shown the decreased electroshock seizure threshold phenotype in the VPA-exposed rat models of ASD (10, 12, 13). This condition was suggested to be related to the excitatory/inhibitory imbalance in the brain (10, 67) through upregulated excitatory processes but reduced GABAergic receptor system (10, 68, 69). The result of the present study, therefore, confirms the previous results and extends the knowledge that increased seizure susceptibility is also true for VPA-exposed mice. Certainly, studying the brains of VPA-exposed mice will be needed to confirm and compare the molecular results found in rats and postmortem human brains. Here, we further showed the potential therapeutic effects of KRG in increasing the seizure threshold in the VPA-exposed mice. With regards to the well-studied GABAergic system reduction in ASD patients and rat models, studying the GABAergic system in the VPA-exposed mice will also be needed for confirmation and elucidation of how KRG achieved its therapeutic mechanism.

The mechanism of actions underlying the therapeutic effect of long-term KRG treatment in the postnatal conditions is highly essential; these steps should be investigated in the near future. In addition, further studies are needed to isolate and identify the specific ginsenosides of KRG that are responsible for the alleviation of various behaviors in VPA-exposed models. In any case, we believe that the postnatal therapeutic effects of KRG provide additional lines of potential options, on top of the growing list of candidate molecules and targets that could treat the core symptoms of ASD even after brain development, which could be more clinically relevant. Whether the therapeutic effects of KRG are mediated by neurochemical or microstructural changes in the brain would be an additional important question to be answered, which could provide hints on the pathophysiological mechanisms of ASD. Finally, this study importantly showed the wide array of benefits in alleviating behavior impairments in animal models of ASD through supplementation of an herbal or nutraceutical product found in KRG.
